# Safety of human papillomavirus vaccines in healthy young women: a meta-analysis of 24 controlled studies

**DOI:** 10.1186/s40780-017-0087-6

**Published:** 2017-07-11

**Authors:** Yukari Ogawa, Hinako Takei, Ryuichi Ogawa, Kiyoshi Mihara

**Affiliations:** 1 Faculty of Pharmacy, Department of Pharmaceutical Sciences, Musashino University, 1-1-20 Shinmachi Nishitokyo-shi, Tokyo, 202-8585 Japan; 2 Department of Pharmacotherapy, Meiji Pharmaceutical University, 2-522-1 Noshio, Kiyose-shi, Tokyo 204-8588 Japan

**Keywords:** HPV vaccine, Cervical cancer, Solicited local symptoms, Solicited systemic symptoms, Safety, Meta-analysis

## Abstract

**Background:**

Human papillomavirus (HPV) vaccines have been shown to be effective for the eradication of HPV and prevention of cervical cancer. However, the number of women who receive HPV vaccinations has decreased over the last several years in Japan, due to concerns about adverse reactions associated with the vaccines. We evaluated the safety of three types of HPV vaccines separately in young women and the difference in the risk of adverse reactions between HPV and other vaccines by conducting a meta-analysis.

**Methods:**

Primary literature was retrieved from MEDLINE, the Cochrane Central Register of Controlled Trials, and Japana Centra Revuo Medicina. Prospective controlled studies with participants consisting exclusively of healthy women who received bivalent, quadrivalent, or 9-valent HPV (2vHPV, 4vHPV or 9vHPV) vaccines were included. Primary safety outcome was the incidence of solicited local and systemic symptoms, and unsolicited symptoms. When two or more studies were found for the same analysis, a meta-analysis was applied.

**Results:**

A total of 24 controlled studies from 22 articles were included in our study. Of the 24 studies, 16 were placebo-controlled and eight were active-controlled (different HPV vaccine or hepatitis vaccine). Average ages of the participants ranged from 12 to 37 years. A significantly higher incidence of solicited local symptoms was observed following injection of HPV vaccines (2vHPV and 4vHPV) compared to placebo, but there was no difference between HPV vaccines [risk ratio (RR) for 2vHPV: 1.25, 95% confidence interval (CI): 1.09 to 1.43, RR for 4vHPV: 1.16, 95% CI: 1.11 to 1.20]. The incidence of solicited systemic symptoms was not different between HPV vaccines and placebo (RR: 1.04, 95% CI: 0.99 to 1.09). The incidence of unsolicited symptoms was significantly higher for 2vHPV vaccine compared to placebo (RR: 1.28, 95% CI: 1.01 to 1.63), but was not significantly different between 2vHPV and hepatitis B vaccines.

**Conclusions:**

HPV vaccines had significantly higher risk of any injection site symptom compared to placebo or other vaccines (hepatitis A and B vaccines), and the incidence of solicited local symptoms was no difference between 2vHPV vaccination and 4vHPV vaccination. However, the most adverse reactions were transient.

## Background

Cervical cancer is the fourth most common cancer among women worldwide [1, 2] and a major world health problem for women. The estimated number of cervical cancer cases in 2012 was 528,000, and the number of deaths was 266,000 [3] in the world. In Japan, 2813 persons died from the disease in 2015, with crude mortality rate of 4.4 per 100 thousand population [4]. Both incidence and mortality of cervical cancer in young women have been reported to increase over the last 20 years [4]. Furthermore, it is forecasted that 10,600 to 12,600 cases of cervical cancer will be diagnosed annually in 2015 through 2029 in Japan, and 2600 to 2800 will die from the disease [5]. Persistent HPV infection is the most important factor for the development of cervical cancer [6, 7]. Although most of the patients who are infected by human papillomavirus (HPV) present no symptoms, long-standing HPV infection may lead to development of cervical cancer in women.

Of the 15 oncogenic HPV types identified, HPV-16 and HPV-18 account for nearly 70% of all invasive cervical cancer cases worldwide, with HPV-45, HPV-33 and HPV-31 accounting for approximately 10% of reported cases [8]. HPV-16 and HPV-18 also account for 50.3% of all invasive cervical cancer cases in Japan [9]. Since HPV infections are transmitted through sexual contact, girls aged 9 to 14 years are the primary target of HPV vaccination in most of the countries. A clinical trial has shown that prophylactic HPV vaccination is highly efficacious for the prevention of HPV infection and the associated cervical intraepithelial neoplasia [10]. Among three internationally marketed HPV vaccines [bivalent HPV (2vHPV), quadrivalent HPV (4vHPV), and 9-valent HPV (9vHPV) vaccines], 2vHPV and 4vHPV are now being marketed in Japan.

In 2009, the World Health Organization (WHO) issued the first position paper on the inclusion of routine HPV vaccination in national immunization programs [11]. In Japan, financial support from the government was initiated in 2011, and the Ministry of Health, Labour, and Welfare (MHLW) started to promote HPV vaccination in April 2013. However, in May and June 2013, Japanese news media distributed a concern regarding the potential of the vaccine to cause undesirable reactions such as chronic pain, together with sensational coverage of other negative information. In June 2013, MHLW withdrew the recommendation of HPV vaccination [12]. The news of adverse events associated with HPV vaccination strongly affected the vaccination rate in Japan [13], with a substantial decrease in 2013 compared to 2012 [14].

A few meta-analyses have reported the safety of HPV vaccines regarding solicited local and systemic symptoms to date [15, 16]. However, none of the previous meta-analyses evaluated the safety in relation to types of HPV vaccine (i.e., 2vHPV, 4vHPV, and 9vHPV), and these studies compared the safety with combined controls (i.e., placebo and active controls). Therefore, we performed a meta-analysis to re-evaluate the safety of three types of HPV vaccines separately and to evaluate whether the frequency of adverse reactions after HPV vaccination is higher than that after other vaccinations such as hepatitis vaccines in healthy young women, using controlled studies published to date and unpublished studies including Japanese studies.

## Methods

### Data sources and searches

We searched MEDLINE (1966 to February, 2017), the Cochrane Central Register of Controlled Trials (issue 7, 2016), and Japana Centra Revuo Medicina (1981 to February, 2017). We combined the MeSH terms or text words of “papillomavirus vaccine” and “adverse reactions”. We included original research articles written in either English or Japanese. In addition, we manually searched the reference lists of all the selected studies and related articles. Additionally, we reviewed the Pharmaceuticals and Medicines Devices Agency (PMDA) website [17] for any additional unpublished studies relevant to the topic.

### Study selection

The inclusion criteria of the present study were: (1) prospective controlled studies that evaluated safety (solicited local symptoms, solicited systemic symptoms, and unsolicited symptoms); (2) studies that included only healthy women aged nine years or above; and (3) studies in which participants were administered 2vHPV, 4vHPV, or 9vHPV vaccine. Clinical studies in which more than two vaccinations were performed concomitantly and in which all participants were older than 40 years were excluded. Two investigators (YO and HT) applied the inclusion and exclusion criteria independently. When there was a disagreement between the two investigators, a final decision was made after careful discussion.

### Data extraction

Study design (random allocation, blinded); number of subjects; type of HPV vaccine and control used; country or region in which the study was performed; and incidence of solicited local symptoms, solicited systemic symptoms and unsolicited symptoms were extracted from each study. We defined these symptoms as follows [18–20]: Solicited local symptoms were defined as pain, redness and/or swelling at the injection site observed within 15 days after vaccination. Solicited systemic symptoms were defined as arthralgia, fatigue, fever, gastrointestinal symptoms, headache, rash, urticaria, and/or myalgia observed within 15 days after vaccination. All other undesirable reactions occurring within 30 days after injection of the vaccine were included as unsolicited symptoms. We also extracted the incidence of grade 3 solicited local symptoms defined as pain that prevents normal activity, and redness or swelling at the injection site with a diameter greater than 50 mm [21–23].

Primary outcome in the present meta-analysis was the overall incidence of solicited local and systemic symptoms, and that of unsolicited symptoms after HPV vaccination. Secondary outcomes were all the components of the primary outcome as well as cumulative incidence of grade 3 solicited local symptoms, solicited systemic symptoms, and unsolicited symptoms.

### Assessment of risk of bias in included studies

The quality of each included study was assessed using Cochrane’s risk of bias tool [24]. The tool consists of seven categories: random sequence generation (selection bias), allocation concealment (selection bias), blinding of participants and personnel (performance bias), blinding of outcome assessment (detection bias), incomplete outcome data (attention bias), selective reporting (reporting bias) and other bias. We assessed the risk of bias as low, high, or unclear, and the quality of each study is presented in a “risk of bias” table.

### Data synthesis and analysis

We used risk ratio (RR) as a measure of effect for outcomes. We conducted meta-analysis when data of at least two studies were available, and calculated pooled RRs with 95% confidence intervals (CI) using random-effects methods (Mantel-Haenszel). Inter-study heterogeneity was assessed by I^2^ statistic (I^2^ > 75% indicates substantial heterogeneity) [25]. Data were analyzed by Cochrane Review Manager version 5.3 (The Nordic Cochrane Centre, The Cochrane Collaboration).

## Results

### Study retrieval and characteristics of included studies

**Fig. 1:**
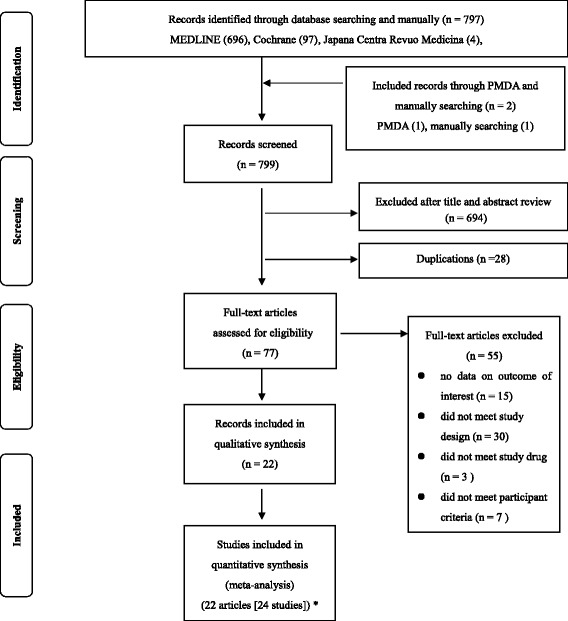
A study selection flow diagram. n: total number of articles. *We included 24 studied from 22 articles because 2articles [26, 47] included 2 studies each. Abbreviations: PMDA, Pharmaceutical Medicines Devices Agency

The primary literature search retrieved 799 articles as follows: MEDLINE (696), Cochrane (97), Japana Centra Revuo Medicina (4), PMDA website (1), manually searching (1). (Fig. 1). Of the 799 articles, 777 were excluded. The reasons for exclusion were: title and abstract review indicated no relevance to our study objective (694), duplications (28), no data on outcome of interest (15), uncontrolled design (30), experimental vaccination (3), and male study or patients complicated with infectious diseases (7). The remaining 22 original research papers [26–47] were included in our analysis. Since two of the papers [26, 47] contained two studies each, we analyzed a total of 24 studies. The number of participants, mean age, HPV vaccine tested, and control of each included study are summarized in Table 1. All the studies were randomized controlled trials. Average ages of participants ranged from 12 to 37 years. Geographic background in the 24 included studies was as follows: Japan (3), other Asian countries (8), Europe (2), Africa (2), United States, and multinational (9). Three studies [28, 29, 45] used HPV vaccines as active controls. The symptoms consisted of the defined adverse reactions after the vaccinations were self-reported in all included studies except for one report [44].

**Table 1:** Summary of studies included in the present meta-analysis

Study	Subjects, n	Age, yr	HPV vaccine (Type of HPV)			Control	Country/Region	Follow-up, months	Reporting of adverse events	Outcome	
			2vHPV	4vHPV	9vHPV					Primary	Secondary
027 Study [26]	841	22.8 ± 2.1		✓		placebo	Japan	30	Self-reported		✓
028 Study [26]	107	12.7 ± 2.4		✓		placebo	Japan	30	Self-reported		✓
Bhatla 2010 [27]	354	28.4 ± 4.91	✓			placebo	India	7	Self-reported	✓	✓
Draper 2013 [28]	198	12 (median)	✓			4vHPV	United Kingdom	Not mention (at least 7 days)	Self-reported		✓
Einstein 2009 [29]	1106	30.5	✓			4vHPV	United States	7	Self-reported		✓
FUTURE II 2007 [30]	12,167	20.0 ± 2.2 (HPV) 19.9 ± 2.1 (Control)		✓		placebo	Multinational	36	Self-reported	✓	✓
Harper 2004 [31]	1113	20 ± 3	✓			placebo	Multinational	27	Self-reported	✓	✓
Kang 2008 [32]	176	16.6		✓		placebo	Korea	7	Self-reported	✓	✓
Kim 2011 [33]	225	22 ± 2.37	✓			placebo	Korea	Not mention (at least 30 days)	Self-reported	✓	✓
Kim 2010 [34]	321	11.9 ± 1.41	✓			hepatitis A vaccine	Korea	7	Self-reported	✓	✓
Konno 2009 [35]	1022	22.5 ± 1.7	✓			hepatitis A vaccine^a^	Japan	7	Self-reported		✓
Lim 2014 [36]	271	24.9 ± 4.02	✓			placebo	Malesia	Not mention (at least 30 days)	Self-reported		✓
Medina 2010 [37]	2067	12.1 ± 1.4	✓			hepatitis A vaccine	Multinational	12	Self-reported	✓	✓
Mugo 2015 [38]	250	-		✓		placebo	Africa	7	Self-reported	✓	✓
Munoz 2009 [39]	3819	34.3 ± 6.3		✓		placebo	Multinational	Not mention (at least 15 days)	Self-reported	✓	
Ngan 2010 [40]	300	26 ± 4	✓			placebo	Hong-Kong	7	Self-reported		✓
Paavonen 2007 [41]	18,644	20.0 ± 3.1	✓			hepatitis A vaccine	Multinational	12	Self-reported	✓	✓
Perez 2008 [42]	6004	19.8 ± 3.0 (HPV) 20.3 ± 2.2 (Control)		✓		placebo	Multinational	7	Self-reported	✓	
Skinner 2014 [43]	5752	37.0 ± 7.2 (HPV) 37.0 ± 7.3 (Control)	✓			placebo	Multinational	48	Self-reported	✓	
Sow 2013 [44]	676	16.9 ± 4.29	✓			placebo	Africa	12	Reported by trained field worker	✓	
Vesikari 2015 [45]	600	12.6 ± 1.9			✓	4vHPV	Europe	7	Self-reported	✓	✓
Villa 2006 [46]	1106	20.0 ± 1.7		✓		placebo	Multinational	7	Self-reported	✓	✓
Zhu-1 2014 [47]	750	13.1 ± 2.43	✓			placebo	China	12	Self-reported	✓	✓
Zhu-2 2014 [47]	1212	35.7 ± 4.99	✓			hepatitis B vaccine	China	12	Self-reported	✓	✓

Primary outcome in our study was the overall incident of solicited local and systemic symptoms, and that of unsolicited symptoms after HPV vaccination. Secondary outcomes was were all the components of the primary outcome as well as cumulative incidence of grade 3 solicited local symptoms, solicited systemic symptoms, and unsolicited symptoms

*Abbreviations: HPV* human papillomavirus, *2vHPV* bivalent human papillomavirus, *4vHPV* quadrivalent human papillomavirus, *9vHPV* 9-valent human papillomavirus

^a^Contained no aluminum as adjuvant

**Fig. 2:**
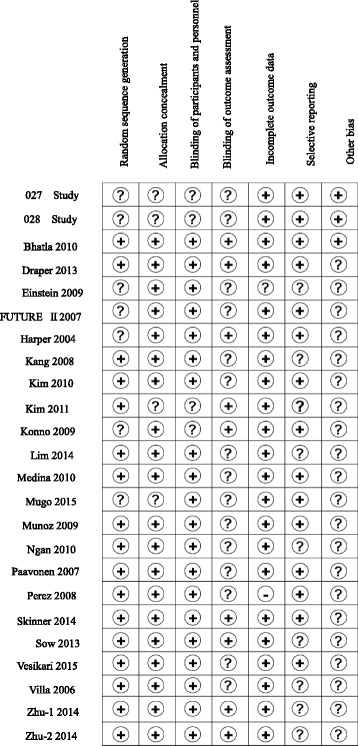
Summary of risk of bias for the 24 studies included in meta-analysis. The judgements were made by two of the study investigators. Symbols: +, low risk of bias; −, high risk of bias;?, bias risk is unclear

Results of risk of bias assessments of the included studies are shown in Fig. 2. Analysis using the Cochrane risk of bias tool indicated an overall low risk of bias. One study [42] had insufficient information on discontinued data. The risks of bias due to inadequate random sequence generation and inadequate allocation concealment were judged to be ‘unclear’ in seven studies [26, 29–31, 35, 38] and four studies [26, 33, 38], respectively.

### Safety of HPV vaccines

**Fig. 3:**
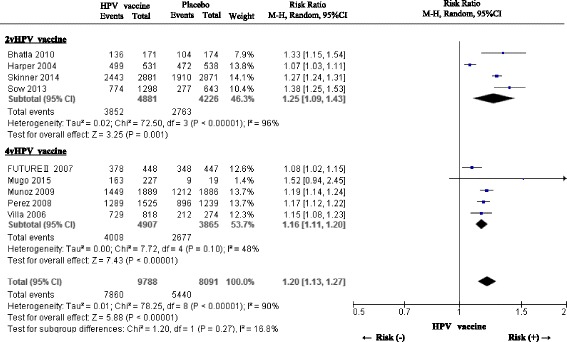
A forest plot of the present meta-analysis on the incidence of solicited local symptoms after administration of HPV vaccines (2vHPV and 4vHPV) compared to placebo injection. Risk ratios (RRs) and 95% confidence intervals (CI) are shown. The analysis was performed using the Mantel-Haenszel method with random effects model. Abbreviations: HPV, human papillomavirus; 2vHPV, bivalent human papillomavirus; 4vHPV, quadrivalent human papillomavirus

**Fig. 4:**
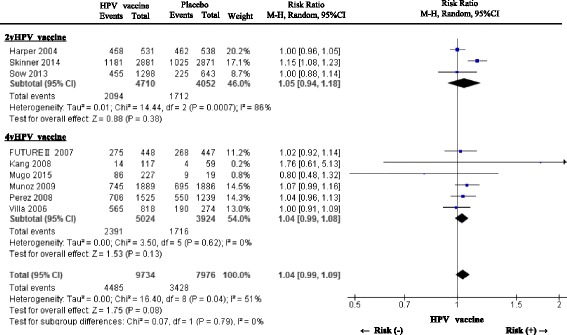
A forest plot of the present meta-analysis on the incidence of solicited systemic symptoms after administration of HPV vaccines (2vHPV and 4vHPV) compared to placebo injection. Risk ratios (RRs) and 95% confidence intervals (CI) are shown. The analysis was performed using the Mantel-Haenszel method with random effects model. Abbreviations: HPV, human papillomavirus; 2vHPV, bivalent human papillomavirus; 4vHPV, quadrivalent human papillomavirus

**Fig. 5:**
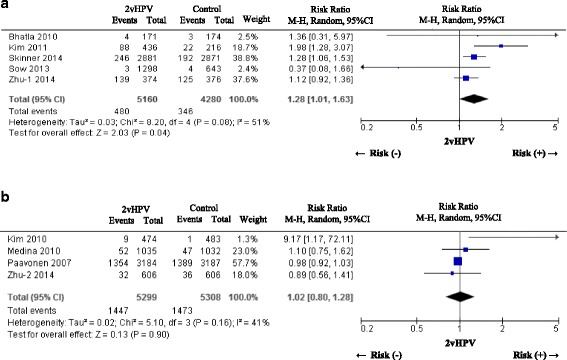
A forest plot of the present meta-analysis on the incidence of unsolicited symptoms after administration of 2vHPV compared to placebo or hepatitis vaccine. **a** 2vHPV versus Placebo, **b** 2vHPV versus Hepatitis vaccine. Risk ratios (RRs) and 95% confidence intervals (CI) are shown. The analysis was performed using the Mantel-Haenszel method with random effects model. Abbreviations: HPV, human papillomavirus; 2vHPV, bivalent human papillomavirus

1. *Solicited local symptoms*
  We analyzed nine placebo-controlled studies (four 2vHPV vaccine [27, 31, 43, 44] and five 4vHPV vaccine studies [30, 38, 39, 42, 46]) for the primary safety outcome. A meta-analysis showed a significantly higher incidence of any solicited local symptom in the pooled HPV vaccine (2vHPV and 4vHPV) group compared to the placebo group (overall RR: 1.20, 95% CI: 1.13 to 1.27) (Fig. 3). There was no difference between types of HPV vaccine (pooled RR for 2vHPV: 1.25 [1.09 to 1.43], pooled RR for 4vHPV 1.16 [1.11 to 1.20]).
  All HPV vaccines were associated with significantly increased incidence in all components of solicited local symptoms (pain, redness and swelling at the injection sites) (Table 2). In five hepatitis vaccine-controlled studies, the incidence of pain, redness, and swelling was significantly higher in the 2vHPV vaccine group than in the hepatitis vaccine group (pooled RRs [95% CIs] for pain: 1.80 [1.29 to 2.51], redness 1.77 [1.55 to 2.02], and swelling 2.57 [2.19 to 3.02]). Our study found no hepatitis vaccine-controlled study of 4vHPV vaccine. Two studies mentioned that there were subjects discontinued due to serious injection-site symptom. In one study, one subject (less than 0.1%) in the placebo group discontinued due to hypersensitivity. In another study, five subjects (0.3%) in 4vHPV group withdrew due to solicited local symptoms. In others, adverse reactions were transient, and there was no participant who discontinued the study schedule owing to the solicited local symptoms.
2. *Grade 3 solicited local symptoms*
  We gathered information on grade 3 solicited local symptoms from the 2vHPV vaccine studies (three hepatitis vaccine-controlled studies [34, 37, 41] and two placebo-controlled studies [27, 40]) (Table 3). The 2vHPV vaccine showed a significantly higher incidence of pain compared to placebo, with RR [95% CI] of 6.58 [3.03 to 4.30]. In addition, the 2vHPV vaccine was associated with higher incidence of grade 3 symptoms compared to hepatitis vaccine, with pooled RRs [95% CIs] of 4.49 [3.02 to 6.68] for pain, 6.30 [1.97 to 20.16] for redness, and 5.04 [3.21 to 7.91] for swelling. The durations of the symptoms, however, were similar in 2vHPV and control groups. Three of the five studies [34, 40, 41] reported that the symptoms lasted no longer than 4–5 days in both groups, and the remaining two studies did not report the duration of symptoms [27, 37]. Only one study tested 4vHPV [38] reported grade 3 pain, and there was no significant difference in the pain compared with the placebo arm (RR: 0.50 [0.06 to 3.96]).
3. *Solicited systemic symptoms*
  We analyzed nine placebo-controlled studies (three 2vHPV vaccine [31, 43, 44] and six 4vHPV vaccine studies [30, 32, 38, 39, 42, 46]). There was no significant difference when comparing 2vHPV or 4vHPV vaccine with placebo (Fig. 4). Comparing to placebo, 2vHPV vaccine group showed significantly higher RRs of fatigue, gastrointestinal symptoms, and myalgia but not in arthralgia, fever, rash, urticarial, and headache (Table 4). On the other hand, analysis of five hepatitis vaccine-controlled studies showed that the incidence of all components of solicited systemic symptoms except for gastrointestinal symptoms and myalgia was significantly higher in the 2vHPV vaccine group than in the hepatitis vaccine group (Table 4). Gastrointestinal symptoms and headache were components of interest in a meta-analysis of 4vHPV vaccine, and no significant differences were observed compared to placebo.
4. *Unsolicited symptoms*
  Five placebo-controlled studies [27, 33, 43, 44, 47] and four hepatitis vaccine-controlled studies [34, 37, 41, 47] were available to assess the risk of unsolicited symptoms associated with 2vHPV vaccine. Three studies [34, 37, 41] used hepatitis A vaccine and one study [47] used hepatitis B vaccine as control. The 2vHPV vaccine had a slightly but significantly higher risk of unsolicited symptoms compared to placebo (pooled RR [95% CI]: 1.28 [1.01 to 1.63]), but there was no difference between 2vHPV vaccine and hepatitis vaccine (pooled RR [95% CI]: 1.02 [0.80 to 1.28]) (Fig. 5). The most frequently reported unsolicited symptoms were infections such as upper respiratory tract infection, nasopharyngitis, and dizziness. There was no data regarding 4vHPV vaccine.
5. *4vHPV versus 2vHPV or 9vHPV*
  We included three head-to-head comparison studies (4vHPV vaccine versus 2vHPV vaccine in two studies [28, 29], 4vHPV vaccine versus 9vHPV vaccine in one study [45]). Regarding solicited local symptoms, the 4vHPV vaccine had apparently lower risk of developing pain and redness than the other two HPV vaccines, but the differences did not reach statistical significance (pooled RR [95% CI] for pain: 0.89 [0.75 to 1.05] and redness: 0.70 [0.46 to 1.07]). On the other hand, the 4vHPV vaccine was significantly lower risk of developing swelling (pooled RR [95% CI]: 0.71 [0.57 to 0.88]). Regarding solicited systemic symptoms, 4vHPV vaccine had a significantly lower risk of fatigue compared to 2vHPV vaccine (pooled RR: 0.82, 95% CI: 0.73 to 0.92) (Table 5).

**Table 2:** Pooled risk ratios of solicited local symptoms

Comparison	Studies, n	Subjects, n	Pooled RR [95% CI]	Reference
Pain				
2vHPV vaccine vs Hepatitis vaccine^a^	5	15,471	1.80 [1.29, 2.51]	[34, 35, 37, 41, 47]
2vHPVvaccine vs Placebo	7	5315	1.34 [1.15, 1.57]	[27, 31, 33, 36, 40, 44, 47]
4vHPVvaccine vs Placebo	6	3474	1.17 [1.10, 1.24]	[26, 30, 32, 38, 46]
Redness				
2vHPV vaccine vs Hepatitis vaccine^a^	5	15,471	1.77 [1.55, 2.02]	[34, 35, 37, 41, 47]
2vHPV vaccine vs Placebo	6	3374	1.77 [1.52, 2.07]	[27, 31, 33, 36, 40, 47]
4vHPV vaccine vs Placebo	2	1055	1.47 [1.18, 1.82]	[26]
Swelling				
2vHPV vaccine vs Hepatitis vaccine^a^	5	15,471	2.57 [2.19, 3.02]	[34, 35, 37, 41, 47]
2vHPV vaccine vs Placebo	7	5315	2.27 [1.78, 2.91]	[27, 31, 33, 36, 40, 44, 47]
4vHPV vaccine vs Placebo	4	2403	1.83 [1.50, 2.22]	[26, 38, 46]

*Abbreviations: HPV* human papillomavirus, *2vHPV* bivalent human papillomavirus, *4vHPV* quadrivalent human papillomavirus, *RR* risk ratio, *CI* confidence interval

^a^Four studies [34, 35, 37, 41] used hepatitis A and one study [47] used hepatitis B vaccine as control

**Table 3:** Pooled risk ratios of grade 3 solicited local symptoms

Comparison	Studies, n	Subjects, n	Pooled RR [95% CI]	Reference
Pain				
2vHPV vaccine vs Hepatitis vaccine^a^	3	13,237	4.49 [3.02, 6.68]	[34, 37, 41]
2vHPV vaccine vs Placebo	2	645	6.58 [3.03, 4.30]	[27, 40]
Redness				
2vHPV vaccine vs Hepatitis vaccine^a^	3	13,237	6.30 [1.97, 20.16]	[34, 37, 41]
2vHPV vaccine vs Placebo	2	645	1.62 [0.20, 13.07]	[27, 40]
Swelling				
2vHPV vaccine vs Hepatitis vaccine^a^	3	13,237	5.04 [3.21, 7.91]	[34, 37, 41]
2vHPV vaccine vs Placebo	2	645	2.52 [0.58, 10.97]	[27, 40]

Grade 3 solicited local symptoms define as pain that prevents normal activity, and redness or swelling at the injection site with a diameter greater than 50 mm

*Abbreviations: HPV* human papillomavirus, *2vHPV* bivalent human papillomavirus, *RR* risk ratio, *CI* confidence interval

^a^All studies [27, 34, 37, 40, 41] used hepatitis A as control

**Table 4:** Pooled risk ratios of solicited systemic symptoms

Comparison	Studies, n	Subjects, n	Pooled RR [95% CI]	Reference
Arthralgia				
2vHPV vaccine vs Hepatitis vaccine	5	15,470	1.38 [1.12, 1.70]	[34, 35, 37, 41, 47]
2vHPVvaccine vs Placebo	6	4246	1.03 [0.40, 2.69]	[27, 33, 36, 40, 44, 47]
Fatigue				
2vHPV vaccine vs Hepatitis vaccine^a^	5	15,470	1.13 [1.05, 1.22]	[34, 35, 37, 41, 47]
2vHPV vaccine vs Placebo	7	5315	1.23 [1.04, 1.44]	[27, 31, 33, 36, 40, 44, 47]
Fever				
2vHPV vaccine vs Hepatitis vaccine^a^	5	15,470	1.17 [1.05, 1.29]	[34, 35, 37, 41, 47]
2vHPV vaccine vs Placebo	7	5315	1.11 [0.95, 1.28]	[27, 31, 33, 36, 40, 44, 47]
Gastrointestinal symptoms				
2vHPV vaccine vs Hepatitis vaccine^a^	5	15,470	1.03 [0.97, 1.10]	[34, 35, 37, 41, 47]
2vHPV vaccine vs Placebo	7	10,796	1.46 [1.06, 2.02]	[27, 31, 33, 40, 43, 44, 47]
4vHPV vaccine vs Placebo	2	1990	0.92 [0.77, 1.11]	
Rash				
2vHPV vaccine vs Hepatitis vaccine^a^	5	15,470	1.43 [1.15, 1.77]	[34, 35, 37, 41, 47]
2vHPV vaccine vs Placebo	7	10,796	1.26 [0.80, 1.99]	[27, 31, 33, 40, 43, 44, 47]
Urticaria				
2vHPV vaccine vs Hepatitis vaccine^a^	5	15,470	1.21 [1.05, 1.39]	[34, 35, 37, 41, 47]
2vHPV vaccine vs Placebo	6	9727	1.04 [0.52, 2.08]	[27, 33, 40, 43, 44, 47]
Headache				
2vHPV vaccine vs Hepatitis vaccine^a^	5	15,470	1.09 [1.01, 1.18]	[34, 35, 37, 41, 47]
2vHPV vaccine vs Placebo	7	10,796	1.62 [0.83, 3.19]	[27, 31, 33, 40, 43, 44, 47]
4vHPV vaccine vs Placebo	4	2396	1.01 [0.85, 1.19]	[26, 38, 46]
Myalgia				
2vHPV vaccine vs Hepatitis vaccine^a^	5	15,470	1.07 [0.72 1.58]	[34, 35, 37, 41, 47]
2vHPV vaccine vs Placebo	6	9727	1.54 [1.31, 1.81]	[27, 33, 40, 43, 44, 47]

*Abbreviations: HPV* human papillomavirus, *2vHPV* bivalent human papillomavirus, *4vHPV* quadrivalent human papillomavirus, *RR* risk ratio, *CI* confidence interval

^a^Four studies [34, 35, 37, 41] used hepatitis A and one study [47] used hepatitis B vaccine as control

**Table 5:** Comparison of 4vHPV versus other HPV vaccines regarding solicited local and systemic symptoms

Components	Studies, n	Subjects, n	Pooled RR [95% CI]	Reference
Pain	3	1845	0.89 [0.75, 1.05]	[28, 29, 45]
Redness	2	1246	0.70 [0.46, 1.07]	[28, 29]
Swelling	3	1845	0.71 [0.57, 0.88]	[28, 29, 45]
Fatigue	2	1250	0.82 [0.73, 0.92]	[28, 29]

Other HPV vaccines stand for 2vHPV and 9vHPV

Two studies [28, 29] compare 4vHPV with 2vHPV and one study [45] compares 4vHPV with 9vHPV

*Abbreviations: HPV* human papillomavirus, *2vHPV* bivalent human papillomavirus, *4vHPV* quadrivalent human papillomavirus, *RR* risk ratio, *CI* confidence interval

## Discussion

The 2vHPV vaccine and 4vHPV vaccine were licensed in 2009 and 2011, respectively, in Japan. The 9vHPV vaccine has been approved by the Food and Drug Administration in 2014 but not yet in Japan. We focused on the safety of 2vHPV and 4vHPV vaccines in the present study because MHLW has withdrawn the recommendation of HPV vaccination for adolescent women aiming to prevent cervical cancer, due to a concern over adverse reactions. Our meta-analysis summarized evidence-based safety profiles of HPV vaccines based on controlled studies including unpublished studies. The five major findings from our study are as follows. (1) Solicited local symptoms are more frequent after HPV vaccinations than after both placebo injection and hepatitis vaccination. (2) The risk of developing solicited local symptoms is no difference between 2vHPV vaccine and 4vHPV vaccine. (3) Most of observed solicited local symptoms such as pain, redness and swelling, even grade 3 symptoms, are transient. (4) The incidence of most of the solicited systemic symptoms is higher after HPV vaccination than after placebo injection or hepatitis vaccination. (5) Unsolicited symptoms after HPV vaccination appear to be more frequent compared to placebo injection, but similar to hepatitis vaccination.

There is a difference in pharmaceutical property between 2vHPV and 4vHPV vaccines. The 2vHPV vaccine contains the adjuvant system (AS)-04 composed of 3-O-desacyl-4′-monophosphoryl lipid A (MPL) and aluminum salt (500 μg as aluminum). On the other hand, the 4vHPV vaccine contains only aluminum salt (225 μg as aluminum). AS-04 is one of the new-generation adjuvants licensed for use in human vaccines, and has been developed to enhance vaccine-induced immune response [48, 49]. MPL is derived from chemical modification of the potent immunomodulatory lipopolysaccharide (LPS) of *Salmonella minnesota*. Humans are regularly exposed to LPS produced by naturally existing bacteria because many bacterial species contain LPS as a major component of the bacterial cell wall [50]. AS-04 is contained only in 2vHPV vaccine among marketed HPV vaccines in Japan. On the other hand, an AS-04–containing hepatitis B vaccine (Fendrix®, GlaxoSmithKline Biologicals) has shown non-inferiority in safety (including solicited local symptoms and systemic symptoms) compared to a standard hepatitis B vaccine that does not contain AS-04 [51]. Osmotic pressure and pH of HPV vaccines are similar to other subcutaneous vaccines (2vHPV: pH 6.0–7.0, osmotic pressure ratio 1.0; 4vHPV: pH 5.7–6.7, osmotic pressure ratio 2.0). The difference in pharmaceutical property may not affect the safety profile in our meta-analysis.

Mixing aluminum salts in vaccines is a conventional method of non-specific proinflammatory augmentation of immune response and has been widely used as an adjuvant for vaccines [52]. According to a meta-analysis on adverse events after administration of aluminum-containing vaccines to children aged 10 to 16 years, there is no significant association between exposure to aluminum–containing vaccines and local induration/swelling and local pain lasting up to 14 days [53]. Furthermore, aluminum-containing vaccines other than HPV vaccines (including influenza, pneumococcal, and hepatitis B vaccines) have been widely used in clinical practice, and the amount of aluminum in these vaccines ranged from 110 to 500 μg. All included studies except for one study [35] contained aluminum in control group, however, a sensitivity analysis showed there was no discrepancy after removal of the study.

Racial difference may contribute to harmful responses to vaccines. A meta-analysis for safety of HPV vaccines in Asian population concluded that HPV vaccine (2vHPV or 4vHPV) had slightly higher risk of solicited local and systemic symptoms compared to controls (placebo or hepatitis vaccine) [16]. In our subgroup analysis, Asian population tends to have a slightly higher incidence of solicited local symptoms such as pain and swelling after 2vHPV vaccine compared to non-Asian population (pooled RR [95% CI] for pain in Asians and non-Asians: 1.37 [1.30 to 1.45] and 1.24 [0.81 to 1.91], pooled RR [95% CI] for swelling: 2.62 [2.03, 3.40] and 1.62 [1.33 to 1.98], respectively, compared to the respective placebos). On the other hand, pooled RRs [95% CI] for pain after 4vHPV vaccination were 1.23 [1.14 to 1.31] and 1.12 [1.06, 1.19] in Asian and non-Asian populations, respectively. To our knowledge, there is no study that investigates racial difference in incidence of solicited local and systemic symptoms. Further research is required to explain the racial difference.

There are some limitations in our study. First, our study did not focus on complex regional pain syndrome and chronic pain as pain-related symptoms after HPV vaccination. Second, we did not evaluate the efficacy of HPV vaccines systematically. However, many reports have shown the preventive efficacy of HPV vaccines [26–47]. The prevalence of HPV among women aged 14 to 19 years has decreased by 64% since vaccination was introduced a decade ago in the United States [54]. Third, adverse reactions after vaccination were self-reported in all included studies except for one report [44]. We performed a sensitivity analysis and confirmed that the removal of the report did not alter our results and conclusion.

## Conclusions

Our meta-analysis revealed that HPV vaccines had significantly higher risk of any injection site symptom compared to placebo or other vaccines (hepatitis A and B vaccines), and the incidence of solicited local symptoms was no difference between 2vHPV vaccination and 4vHPV vaccination. However, the most adverse reactions were transient.

## Abbreviations

2vHPV: Bivalent HPV
4vHPV: quadrivalent HPV (type 6, 11, 16, and 18)
9vHPV: 9-valent HPV (type 6, 11, 16, 18, 31, 33, 45, 52, and 58)
AS: Adjuvant system
CI: Confidence interval
HPV: Human papillomavirus
LPS: Lipopolysaccharide
MHLW: The Ministry of Health, Labour, and Welfare
MPL: 3-O-desacyl-4′-monophosphoryl lipid A
RCT: Randomized controlled trial
RR: Risk ratio
WHO: The World Health Organization
